# Service Use and Resilience among Adolescents Living with HIV in Blantyre, Malawi

**DOI:** 10.5334/ijic.5538

**Published:** 2021-11-01

**Authors:** Blessings N. Kaunda-Khangamwa, Innocent Maposa, Moffo Phiri, Kennedy Malisita, Emmanuel Mtagalume, Lalio Chigaru, Alister Munthali, Effie Chipeta, Sam Phiri, Lenore Manderson

**Affiliations:** 1 School of Public Health, The University of the Witwatersrand, Johannesburg, South Africa; 2 The School of Public Health and Family Medicine, University of Malawi, College of Medicine, Blantyre, Malawi; 3 The Malaria Alert Centre, University of Malawi, College of Medicine, Blantyre, Malawi; 4 Umodzi Family Centre, Blantyre, Malawi; 5 Centre for Social Research, University of Malawi, Chancellor College, Zomba, Malawi; 6 Centre for Reproductive Health, University of Malawi, College of Medicine, Blantyre, Malawi; 7 Lighthouse Trust, Lilongwe, Malawi; 8 School of Social Sciences, Monash University, Australia; 9 Institute at Brown for Environment and Society, Brown University, Providence RI, USA

**Keywords:** adolescents living with HIV, adolescent-centred approaches, Malawi, resilience, service use, teen-club clinic

## Abstract

**Introduction:**

Adolescents living with HIV (ALHIV) experience social and health challenges that warrant the provision of services and relational support to build resilience. Little is known about how social, community and health services help. We examine formal and alternative service use by and resilience of ALHIV participating in an enhanced teen-club clinic (TCC) programme.

**Description:**

TCC is an adolescent-centred differentiated care model offering a ‘one-stop-shop’ for HIV/Sexual and Reproductive Health (SRH) services to ALHIV. A survey was conducted with 406 ALHIV to determine frequency of use and satisfaction with services. In addition, we conducted 26 in-depth interviews with ALHIV, 12 group discussions with 144 caregivers, and observations of workshops held for 35 health workers to capture multiple perspectives on service use and relational support systems for adolescent’s wellbeing.

**Discussion:**

About 70% of ALHIV were concurrently clients of three or more services. The multi-method analysis showed variations on risks, range of services, frequency of use and satisfaction. Interview data reflected complex factors influencing access to formal services, and caregivers and adolescents also sought alternative care from spiritual and traditional healers.

**Conclusion:**

Adolescent centred-approaches have the potential to enhance resilience promoting resources and outcomes. A multi-sectoral approach to service use and provision is critical to inform adolescent intervention programs and wellbeing.

## Introduction

Adolescents experience high levels of risk associated with living with HIV, compounded by other chronic conditions, abuse, violence, family dysfunction, and community danger. These indicate the need for accessible services [1,2,3,4] including social welfare, juvenile justice, education and physical and mental health services [5,6,7]. Existing studies identify an association between satisfaction with services and resilience and functional outcomes, including better decision making, avoiding high-risk behavior, competence, connections to others, empowerment and continued service use [8,9]. Exploring the interaction between risks, services and resources is key to finding better interventions to support ALHIV.

Differentiated Service Delivery (DSD) is a patient-centred framework, developed by WHO and adapted at the country level to meet the needs of specific populations to reduce health systems inefficiencies [10,11,12]. The WHO’s (2015) *Global Strategy on Integrated People Centred Health Services* advocates for all persons to receive resources and skills for informed decision making on their health and wellbeing [13]; while the Sustainable Development Goals pledge to “leave no-one behind” in social and health initiatives to end AIDS by 2030, and to put adolescents at the centre of the services they need to survive and thrive [14,15]. There is increasing evidence, however, that ALHIV are not benefiting from service provision gains in response to global or national initiatives and agendas, including Malawi [14,16]. For ALHIV, the enhanced teen-club clinic (TCC) model of DSD has gained momentum since 2016 to ensure adolescent-centred HIV care [17]. In Malawi and South Africa, studies of TCCs have identified the role of community-based professionals such as mentors, nurses and psychologists [18,19] in fostering resilience, including supporting the retention of ALHIV on anti-retroviral treatment (ART) programmes [20] and assisting HIV negative adolescents in accessing contraception and antenatal care [21,22].

Formal services for young people facing adversities, such as child welfare, mental health services, alcohol and drug services and juvenile justice, can facilitate positive outcomes [23,24]. Informal and alternative services including traditional rituals do not predict better psychosocial outcomes [25,26], although questions remain about cultural context, adolescents’ choices and use of opportunities to support resilience. We know little of ALHIV service-use and alternative care resources and supports.

## Problem statement

Satisfaction with adolescent-centred services is associated with relationship building, comprehensiveness of interventions, empowerment and respectful engagement with adolescents [8,27]. Previous studies in the south and east Africa show mixed evidence of access and provision of services, adolescents experiences, health workers and caregivers involvement on institutional service use among adolescents [28,29,30,31]. In Malawi, adolescent girls and young women (AGYW) receive public health and social service support for HIV prevention through the Determined, Resilient, Empowered, AIDS-free, Mentored, and Safe (DREAMS) package [27] to ensure wellbeing. The social ecology and supports surrounding vulnerable adolescents are critical, but there has been little attention to service-use satisfaction and resilience. Enhancing promotive factors and service use has the potential to motivate adolescents to do well and to allow their voices to be heard so that they are able to make informed decisions to achieve important everyday goals [15]. Ensuring that service provision is contextually and culturally responsive is key because ALHIV in poor resource settings are most likely to use services when they are meaningful and respectful of context [32,33]. In the research on which we draw for this article, we ask what the perceptions are of resilience-promoting resources among ALHIV. The hypothesis is that greater service use and satisfaction with service will result in high resilience scores and functional outcomes such as pro-social behaviours, interaction with family, peers, school, clinic and access to other institutions.

## Description of the care practice: Teen-club clinics

The Malawi health system is decentralised as a strategy to empower and facilitate central and district hospitals and community ownership, governance and participation in delivering the essential health package [34]. Youth policies and strategies in Malawi advocate for multisectoral and progressive policy approach on SRH for young people to ensure continued support for information, access to care, services for empowerment, development and social wellbeing [35,36]. The structure for adolescent health services in Malawi falls into four levels: the community, primary, secondary and tertiary levels.

For ALHIV, new models of care are provided, such as the DSD, also known locally as the Saturday teen-club clinic/TCC [37,38]. The model offers adolescents with patient-centred care and interventions, which are free at the point of access. The TCC is managed under a Umodzi Family Centre- a centre of excellence in Blantyre, which provides comprehensive range of services (high quality HIV testing, treatment and care, tuberculosis screening, and targeted reproductive health services, psycho-social support and capacity building) for people living with HIV [19,38]. Various partners fund the TCC staff, drugs, operations and programmes and resources: the government of Malawi (ministry of health and National Aids Commission) including bilateral (Centers for Disease Control and Prevention-CDC), multi-lateral (Global Fund) agencies and charitable organisations [38].

The TCC support retention and adherence of ALHIV on ART [37,39]. The TCC in Malawi is a one-stop shop which offers comprehensive HIV clinical care and SRH and psychosocial services for ALHIV aged 9–24 years. Services include health education and support, individual and group counselling, regular consultations with ART treatment refills, and targeted services at facility level [40,41]. Group size varies according to discussion topics, gender, age (9–14, 15–19, and 20–24) and setting, with between 20 and 90 participants in given activities. ALHIV attend group meetings with the same health workers (clinicians, nurses, nutritionist and mentors) each time, ensuring follow up throughout the year.

Most meetings occur once every two months at the Umodzi Family Centre (UFC). At each session, ALHIV arrive at the reception, receive their files and proceed to a common area for medical screening (weight, temperature, blood pressure). Health workers and mentors have their own consultation spaces to provide individual support. Group activities include role-playing and health education sessions, with lunch provided and transport costs reimbursed. Health workers and mentors also contribute to a social welfare fund to help ALHIV with transport and school fees.

## Ethical considerations

Ethics approval was provided by The Human Research Ethics Committee (Medical) of the University of the Witwatersrand (M180465) in South Africa and the College of Medicine Research and Ethics Committee (COMREC) (P.04/18/2389) in Malawi. Ethics consideration was recognised at all levels, including study design, data collection, analysis and presentation [42,43].

## Methodology

A dynamic and iterative mixed methods study [43] included: 1) a self-administered questionnaire survey of 406 ALHIV; 2) in-depth interviews with 26 ALHIV; 3) 12 group discussions with 144 caregivers; 4) workshops with 35 health workers attending a two-day workshop, and 6) analysis of the Electronic Medical Records (EMR) of the 406 ALHIV. This paper is part of a larger study reported elsewhere [19,40].

The study took place in the TCC programme of the UFC at Queen Elizabeth Central Hospital (QECH) in Blantyre from 1 November 2018 to 30 June 2019. Stratified sampling was used to identify ALHIV and health workers involved in the UFC ART and TCC programme. Strata were based on HIV status, TCC attendants (full or partial) and non-attendants. ALHIV who presented as severely sick or those who had not disclosed their status to their next of kin were excluded from the study. Sample size calculations reflected the number of adolescents attending clinic (new or established on ART), a strategy used in prior TCC studies [37].

Each of the 406 ALHIV identified a Person Most Knowledgeable (PMK) about their lives, either a health worker, parent or other caregivers. Health workers (19 female, 16 male) included clinicians, a pharmacist, nurses, members of the HIV testing team, mentors, medical records officers, and monitoring and evaluation team members. Of these, 49% were aged 18–29, with three above 50 years. All adolescents nominated PMKs, but the response rate to invitations sent to PMKs was relatively low, with 34% acceptance, due possibly to both stigma and lack of interest. Despite the low turnout, we were able to capture relational perceptions and experiences on service-utilisation among ALHIV. Just over half of the health workers were single (53%), 31% were married, and 16% widowed or divorced. Of the 144 parents and caregivers (mean age 34), 48% were married, 43% single, 9% widowed and the majority were in employment (62%; 28% home-makers, 8% in school, 2% retired).

## Measures

Quantitative data were collected using two pretested structured questionnaires (CYMR-28) [40], the Global Early Adolescent Survey [GEAS]) [44] and medical records from the Electronic Medical Records (EMR) System of the TCC. The EMR provided information on age, gender, age and year at ART initiation, cumulative treatment outcomes and viral load.

The CYRM-28 included survey questions on socio-demographics, risks, service use, frequency, and satisfaction to enable a multi-component approach to explore relationships and patterns between service use and resilience [45]. The GEAS health instrument was developed by WHO, UNFPA and John Hopkins Bloomberg School of Public Health in urban areas of 15 low-income countries [46,47], and included four sub-scales (family, peer, school, and community). The ALHIV completed a GEAS-Health Instrument module with sub-scales [30,36] to determine individual risks and relational and context-based influences of health and wellbeing. Items in instruments and sub-scales were rated on a 5-point Likert scale, ranging from ‘1 = not at all’ to ‘5 = a lot’. Cronbach’s alpha (α) and construct reliability scores for all instruments and subscales were calculated to assess internal consistency of measurements. In general, a good reliability score is >0.60 [48].

The qualitative inquiry elicited multiple perspectives and information on the complex interactions in the TCC, and the significance of these on resilience. We were interested in the tensions and processes for adolescents, including their multi-level social contexts (access to resources, relationships, power, culture) to deepen our understanding of complex and promotive factors related to (dis)engagement with services, relational support, and positive processes and outcomes.

## Instruments and scales

The CYRM-28 captured risks at multiple levels, including the individual, relational and contextual dimensions using sub-scales, with ALHIV responding to statements: ‘I am able to solve problems without harming myself or others’. The α for the CYRM-28 was 0.863. Access to services and utilisation were measured according to quantity/frequency of services used by ALHIV over time; services that they needed but could not get; and satisfaction and meaning with services [32,45].

The service use data collection tool was informed by in-depth interviews and focus group discussions in the pilot study, advisory panel meetings, and caregiver workshops [49,50]. Services included educational support, community teen club, family planning clinic, and traditional healers. A 3-item scale (1 = always, 2 = sometimes and 3 = never) measured adolescents’, health workers’ and caregivers’ responses. Reliability for the measure on the number of services utilised by ALHIV, parents and health workers was 0.709, 0.607 and 0.797, respectively.

To capture service use and frequency, we isolated eight services that ALHIV frequently mentioned under service use (see further below). The overall reliability score was 0.917. The quality and satisfaction of services centred on adolescents’ experiences of frequently nominated services. The service satisfaction questions included 13 items such as ‘I helped choose this service’. The alpha was 0.853.

Fifteen statements measured the influence of peers in adolescents’ lives, for example, ‘How many of your close friends think that it is important to study hard/smoke cigarettes/drink alcohol/use drugs/get tested for HIV, take ART?’ The responses were recorded on a 5-point scale, ranging from ‘1 = All’ to ‘5 = I do not know’. The scores were reversed for analysis. The reliability score was 0.816. At the community level, internal and external risks were captured by focusing on varied forms of supports (7 items) and danger (12 items) using the GEAS. The alpha for internal and external risks sub-scales were 0.824 and 0.859, respectively. The GEAS provided opportunities to assess the role of family and communication, connectedness and monitoring of adolescents. The alpha for this sub-scale was 0.770.

## Data Collection

An invitation was sent by TCC receptionists to ALHIV, parents, and caregivers to attend interviews at the UFC or the Lecture Hall at the College of Medicine. Workshops and guardian sessions were held at intervals and facilitated by the first author to introduce the study and assess their service provision to ALHIV in the past year. ALHIV, health workers and PMKs were encouraged to reflect on their experiences in the past year, and ALHIV use of and/or access to formal and alternative service provision were explored [51].

Two workshops with health workers and 12 guardian sessions with parents/caregivers were held to allow them to inquire and contribute to ALHIV service usage. Focus group discussions were held at weekends to suit adolescents and parents attending school, or working or in business, and to coincide the adolescents presenting for medication refills at the ART clinic. Interviews were held on alternative weekends at the UFC and Malaria Alert Centre seminar rooms and facilitated by the first author to maintain consistency, trustworthiness and confidentiality [19]. Discussions were based on questions around what it meant for ALHIV to do well despite facing challenges in their lives, and to clarify the services ALHIV used.

CYRM and GEAS forms and nomination of PMK were completed by ALHIV in a classroom set-up in a lecture hall, with 20 groups sitting over different days. Each statement on the 50-item questionnaire was read out loud by the first author, and respondents were given a minute to write down their responses for each item. Special support was given to adolescents with hearing or vision problems to ensure that they understood the questions and were able to respond [19].

## Data Analysis

To explore adolescents’ risks, formal and alternative services usage and frequency, and satisfaction to assess resilience, we used a four-step process: 1) multiple response analysis (MRA), 2) composite scoring and reliability, 3) effect sizes through regression, and 4) structural equation modelling (SEM). Analytic procedures were also informed by the resilience manual [50]. MRA computed and summed up health worker, ALHIV, and PMK data to capture the quantity of service provision and utilisation by ALHIV. Composite and average scores were computed by summing up Likert-scale scores (The response options included 1 = never, 2 = needed it but could not get it, 3 = once a month, 4 = twice and 5 = three or more times), and were compared to summarise service use quantities and frequency. Data were summarised using descriptive statistics (mean, SD, ranges and quartiles) to aggregate institutional resources frequently used by ALHIV. Composite scores were used to identify protective resources both proximal and distal to the adolescents [8,52].

For reliability coefficients, alpha was done across services and scores to confirm internal consistency: α = 0.80 denotes good reliability and α = <50 indicated an unreliable construct or dimension [40,53]. Correlation coefficients were recorded to ascertain associations of adolescents and variables ranging from risks, service use, frequency and satisfaction. Correlations of approximately 0.1 indicated a small degree of relation, while 0.3 and 0.5 indicate medium and large degrees of relation, respectively [54]. The SEM involved three processes of exploratory factor analysis (EFA) in testing reliability and internal consistency, and in assessing the direct and indirect effects of the multiple factors in the hypothesised model in Stata v14.2 [55]. Inferential statistics, effect size, regression and SEM followed to show the practical significance and relationship of service use and resilience in practice [56]. Confirmatory factor analysis (CFA) tested the fit and construct validity of the scales. Path analysis confirmed the patterns and relationships between risk factors (personal, family, peer, community internal and external) and service usage, frequency and satisfaction.

For the qualitative data, the authors familiarised themselves with the transcripts and notes made during fieldwork, taking account of themes related to formal and alternative service use. The first author used deductive analysis [51], using Bronfenbrenner’s socio-ecological framework [57] to examine the multi-level links and interactions influencing resilience and functional outcomes [58,59]. ALHIV’s capacities, processes, and interactions related to service use and resilience, were coded under six tiers using Nvivo software 11: 1) individual; 2) family; 3) school; 4) community; 5) institutional; and 6) national (policies and intervention programmes). The key themes included services, service delivery, access to service use, knowledge of services, service use satisfaction, tensions, ‘doing well’, relational support, sense of belonging, and empowerment.

The first author also presented and held intensive discussions on the results and analysis processes with co-authors and the UFC clinical and M&E staff to triangulate study findings and interpretation [51,60]. Quotations were identified to best represent the themes on service use and resilience-related outcomes.

## Results

### ALHIV as multiple service users

The results presented below derive from the initial sample of 406 ALHIV, health workers, and parents and other caregivers. Most ALHIV were multiple service users, characterised by formal or institutional services including ART (338, 83%), TCC (308, 76%), community-based teen-clubs (308, 76%), Youth Friendly Health Service (YFHS) (276, 68%) and schools (244, 60%). Alternative services included traditional and spiritual supports to respond to health-related and social vulnerabilities that young people experience in adolescence. Service use alone had a mixed impact on resilience. Satisfaction with the TCC significantly influenced functional outcomes such as community-based service use, better decision making, and fair viral load results: 273 (67%) were suppressed, 92 (23%) were not suppressed, and 41 (10%) reported as missing results. Enhanced TCC programmes and support from health workers impacted on ALHIV resilience-related outcomes. Demographics for ALHIV, including current age, ART initiation year, and cumulative treatment outcomes for the study period are set out in ***Table 1***.

**Table 1:** Summary of demographic characteristics of PLHIV and ALHIV.


VARIABLES			TOTAL

**Persons living with HIV**			33239

**Sex**			

Male			14476

Female			18763

	**NON-TEEN CLUB**	**TEEN CLUB MEMBERS**	

**Sex (10–24 years)**			3945

Male	1447	251	

Female	2000	247	

**ART initiation age**			

<10	3247	350	3597

11 to 14	719	122	841

15 to 19	883	33	916

**Current age**			

10 to 14	1059	237	1296

15 to 19	958	244	1202

20 to 24	1430	17	1447

**Initiation year (10–24 years)**			

1995 to 1999	0	0	0

2000 to 2004	89	1	90

2005 to 2009	1382	62	1444

2010 to 2014	925	50	975

2015 to 2019	1051	54	1105

**Cumulative treatment outcomes (15–19)**			

Transferred out	13	0	13

Retained	185	221	406

Defaulted	758	28	786

Stopped	2	1	3

Died	0	0	0

Total not retained	773	29	802

**VIRAL LOAD**	**NON-TEEN CLUB**	**TEEN CLUB MEMBERS**	**TOTAL**

All ALHIV (10–24)	–	–	35%(n = 3500)*

All adolescents (10–24)	–	90%(n = 498)	

Virologic failure (10–24)	–	24%(n = 447)	21%(n = 1225)

Virologic failure (15–19)	–	59%(n = 106)	35%(n = 252)

Viral load suppression (10–24)	–	75%(n = 447)	79%(n = 1225)

Viral load suppression (15–19)	–	44%(n = 335)	31%(n = 973)

Missing	–	14%(n = 238)	


* Results for viral load for some ALHIV were missing. Source: UFC Electronic Medical Records (EMR) System. Baseline data as of 1 November 2018–30 June 2019.

As observed in response patterns [40], factor structures showed resilience to be multi-dimensional and dynamic across sub-populations. We confirmed factor structures, reliability, correlations, and validity for all measures prior to conducting inferential statistics. Cronbach’s alpha scores indicated good internal reliability across all dimensions, except for a specific service use domain on cultural services (α = 0.59 due to lower response rate), 0.77 (family risk) to 0.92 (service-usage frequency). Health workers and parents/caregivers recorded 0.80 and 0.61 on service use, respectively

Correlations ranged from –0.07 to 0.35, indicating a weak relationship between family and personal risks (0.28), internal community and peer risks (0.31), internal and external risks (0.35), and service frequency and quantity (0.28) (***Table 2***, below). These results suggest the distinctiveness of the factors under assessment as well as variance of responses across ALHIV [58,61].

**Table 2:** Mean, SD, Range and reliability of each factor and inter-correlations among dimensions.


	MEAN	SD	RANGE	1	2	3	4	5	6	7	8	9	10	11

1. Personal risk (ALHIV)	23.4	9.91	7–45	.84*										

2. Family risk	23.8	7.85	7–50	.28	.77*									

3. Peer risk	50.5	8.95	15–75	.12	.15	.82*								

4. Community risk (Internal)	25.5	8.92	10–50	.12	.12	.31	.82*							

5. Community risk (External)	25.5	7.64	7–35	.18	.13	.19	.35	.86*						

6. Service use (quantity)	46.5	5.71	25–54	–.07	.00	.17	.07	.04	.71*					

7. Service use (frequency)	106.0	28.6	55–255	.03	.04	.12	.05	.11	.28	.92*				

8. Service use (satisfaction)	19.5	5.99	0–39	.04	.08	.07	.15	.12	.10	–.14	.85*			

9. Service use (PMK)	46.6	3.68	25–54	–	–	–	–	–	–	–	–	.61*		

10. Service use (HW)	40.6	9.02	0–54	–	–	–	–	–	–	–	–	–	.80*	

Resilience (ALHIV)	118.9	14.9	53–140	–.08	.09	–.04	–.07	.00	.04	.02	–.04	–	–	.86*


* Representing reliabilities for factors (Cronbach’s alpha), no correlation coefficients, (–) missing ratings on different dimension.

Multiple group responses on service nominations using MRA found that service use differed slightly among ALHIV, parents/caregivers and health workers (***Table 3***).

**Table 3:** ALHIV Frequency of service use.


NOMINATED SERVICES	HEALTH SERVICES	FREQUENCY OF SERVICE USE*				

		*ALWAYS*	*SOMETIMES*	*NEVER*	NO RESPONSE	TOTAL

**Sexual & Reproductive services**	Youth-friendly health services clinic	184	92	130	–	406 (68%)

	Ante-natal Clinic	7	7	385	7	399 (3%)

	Maternity	37	20	349	–	406 (14%)

	Labour ward	7	6	382	11	395 (3%)

	Gynaecology	4	1	374	27	379 (1%)

	Emergency Pills	5	1	372	28	378 (1%)

	STI Clinic	85	44	276	1	405 (32%)

	Family planning clinic	3	14	363	26	380 (4%)

	Skin specialist	8	2	369	27	379 (2%)

**HIV Clinic**	Teen club clinic	234	74	98	–	406 (76%)

	ART clinic	302	36	68	–	406 (83%)

**Educational services**	Education Support	159	85	162	–	406 (60%)

**Mental Health**	Mental health support	22	24	360	–	406 (11%)

**Correctional services**	One-stop centre	35	39	331	1	405 (18%)

	Police service	14	22	368	2	404 (9%)

**Community services**	Youth centre	95	115	196	3	403 (52%)

	Community teen club	242	61	102	1	405 (75%)

	Community Outreach Clinic	86	83	237	2	404 (42%)

	Traditional healers	27	44	335	26	380 (27%)


* % of adolescents whoever used the services.

Most parents/caregivers responded that ALHIV were concurrent service users; of these, 318 (78%) accessed HIV-related services, including TCC (112, 30%), ART clinic (100, 27%), YFHS and educational support (40, 11%) and the community-based teen club (25, 7%). Health workers gave similar responses: ART clinic and TCC (29, 16%), ART clinic (26, 15%), community-based teen club (25,14%), YFHS (17, 10%) and educational support (14, 8%). Parents/caregivers reported lower utilisation than health workers of ALHIV for Sexual and Reproductive Health (SRH), Family Planning (FP), Sexual Transmitted Infections (STI), emergency pill, gynaecology care, maternity care, mental health, correctional, traditional healers, and spiritual services. The most common service use reported by ALHIV are provided in ***Table 3***.

Qualitative data on service use varied, as most participants had limited knowledge of services outside the teen club. Most health workers, caregivers and adolescents mentioned failure to refer adolescents to other health-related departments due to time constraints in consultations with young people:

I fail to refer some adolescents to gynaecology [department] because some of them refuse, while others need me to act as their guardian so that they are helped faster. It becomes a challenge due to time constraint (health worker, female).

It is difficult to refer adolescents because of lack of time to walk through the referral process as well as to follow the standard follow-up visits (health worker, male).

Some caregivers and health workers encouraged ALHIV to remain in school rather than present for services, because of the importance placed on education. Caregivers commented on possible ways that they could use rituals and customs such as rite of passage rituals, and involving religious pastors and other spiritual leaders, to improve the lives of their children. Health workers and caregivers were divided on the role of traditional healers, with the former seeing no advantages: “There is no need to send a child to a traditional healer. Sometimes their work is not scientifically proven to heal diseases” (health worker, male). However, caregivers and older adolescents considered traditional medicine to be significant, and some parents and male adolescents used traditional medicines such as *mchape* – a concoction or purgative that cleansed their bodies because they do not menstruate. One mother explained: “I need more spiritual support, the prayers, and sometimes we also use traditional medicine to cleanse her system. As a family, we also encourage her taking ART, but we cannot forget where we come from” (mother, 33 years, to daughter, 17 years). Older boys and girls also showed interest in herbs for sexual function and response.

### Service use, frequency and satisfaction

On average, the most frequently used services on a monthly basis were community services (composite score = 18.5; average score = 2.6), HIV/ART care, and education services. Most ALHIV needed but could not get the following services: SRH, YFHS, mental health, child welfare and cultural services (rite-of-passage ceremonies); most ALHIV reported that they did not need correctional services (***Table 4***). Overall the alpha scores (>0.70) indicate good internal reliability across social and health service dimensions with the exception of cultural services with α = 0.59 due to lower response rate.

**Table 4:** Summary of service use scores and other descriptive statistics.


SERVICE USE FREQUENCY	SCORES	MEAN	SD	MIN–MAX	CRONBACH’S ALPHA

Community services	Composite	18.5	7.11	6–35	0.820

	Average	2.6	1.02	1–5	

HIV/ART care services	Composite	17.7	6.14	4– 40	0.731

	Average	2.2	.77	.5–5	

Educational Services	Composite	12.9	5.23	5–25	0.719

	Average	2.6	1.05	1–5	

Youth-friendly services	Composite	16.9	7.17	8–15	0.814

	Average	1.9	.79	.88–5	

Mental Health	Composite	11.9	4.87	0–35	0.695

	Average	1.7	.69	0–5	

Child welfare	Composite	10.4	5.2	1–35	0.824

	Average	1.49	.74	.14–5	

Correctional services	Composite	7.23	3.10	5–30	0.818

	Average	1.2	.52	.83–5	

Cultural Services	Composite	10.35	3.98	5–24	0.593

	Average	1.7	.66	.83–4	


On service use satisfaction, ALHIV preferred the TCC with which they had a lot of contact. They were satisfied with the quality of services (395 [97%], mean 32.37, SD, 5.93 min = 13 and max = 39). Few adolescents (1%) mentioned the church or community teen-club as providing services satisfactorily. Only four ALHIV reported no access to any service, despite being recruited through the ART clinic. In addition to the TCC providing ART and support, most adolescents mentioned that it provided a conducive environment to make friends or develop an intimate relationship, manage their lives, and access health education and monetary support to pay their school fees.

The mentors [ALHIV role models] and health workers who provide the service are very friendly and value sympathy and empathy (Tiwale, female, 19 years).

I have learnt a lot under the teen club. I appreciate myself more. I interact with others better. I found what I was missing in my life (Timo, female, 15 years).

I stopped doubting myself. I have more confidence and I take my medication (Kemo, male, 16 years).

### Complex paths for ALHIV resilience

**Figure 1:**
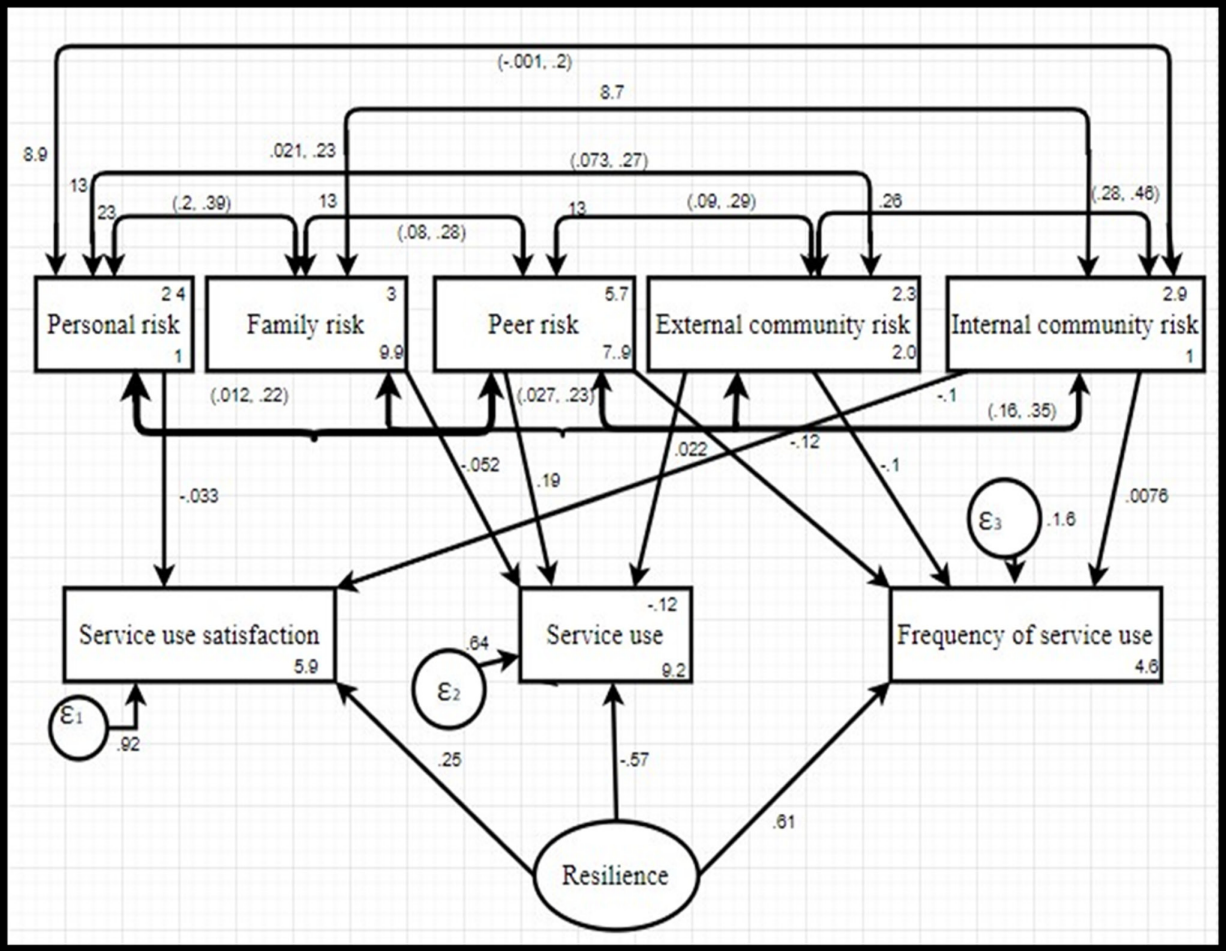
Path analysis model of risks, service use, and resilience for ALHIV.

Structural equation modelling (SEM) and path analysis captured the complex patterns of ALHIV resilience, interacting with personal, family, peer, internal and external community risks and mediating factors (service use, frequency, satisfaction) [62,63] (***Figure 1***). The initial model was a poor fit but was modified by adding a path on internal community risk and service frequency to service satisfaction; and peer risk and external community risk to service frequency. The p-value = 0.525, Root Mean Square Residual Approximation (RMSEA) = .000, Comparative Fit Index (CFI) = 1.000, Tucker-Lewis index = 1.027, standardised root mean squared residual (SRMR) = 0.021.

The paths between risks, service usage, frequency, satisfaction and resilience show the collective strength of relationships of multiple factors for ALHIV resilience. Internal community risks and resilience proved to be statistically significant (p-value < 0.001) with direct effects, implying association and not causation, on service satisfaction. ALHIV facing peer risks and external risks were more likely to use services than youth facing personal risks or internal risks. Peer risks and resilience were also statistically significant, with direct effects on service use frequency. Overall, resilience positively influenced service use frequency as depicted by the standardized coefficient of .61. The higher the resilience, the more often services were used, but the fewer services were used over time. There was no indirect effect of risk dimensions and other variables to overall resilience (***Figure 1***).

### Relationship between risks, service use and resilience

We observed the differential functioning of resilience processes and scores across age and gender, correlation and strength of relationships. The CYRM-28 standard minimum and maximum scores indicate 28 and 140, respectively. In the current study, the minimum and maximum scores were 53 and 140, mean score 118.2. To categorise high and low resilience scores, we used the 50 percentiles as the cut-off, with study participants scoring above 50 defined as having higher resilience. The distribution of resilience scores for the lowest quartile was between 53 and 110, the second quartile 111–123, third 124–130, and fourth 131–140. At 25%, 108 ALHIV scored 110 or lower; at the upper quartile, 75%, 87 scored above 124. According to the resilience research centre manual, the higher the resilience score, the more resilience components/supports are present [50].

Associations between service usage and higher scores of resilience were significant. ALHIV with high resilience scores used more services (p-value < 0.001), with young men having a higher score of service use than girls. A high score in service use frequency and satisfaction with TCC reduced the use of multiple services. Those with primary school education were three times less likely to access services than those not in school; ALHIV not in school and those aged 17–19 used more services over time. Those with a suppressed viral load had a lower score of service use (***Table 5***).

**Table 5:** Regression on resilience, service use, viral load suppression and demographic factors.


VARIABLE	COEFF.	P-VALUE	95% CONF. INTERVAL	

			LOWER	UPPER

**Resilience level**				

High resilience	Ref	–	–	–

Lower resilience score	.92	0.043	.03	1.82

Service use frequency	–.56	0.000	–.72	–.04

Service use satisfaction	–.05	0.170	–.13	.02

**Gender**				

Female	Ref	–	–	–

male	.61	0.18	–.30	1.50

**Education**				

No education	Ref	–	–	–

Education (Primary)	–3.12	0.000	–4.81	–1.56

Education (Secondary)	–1.82	0.02	–3.31	–.33

**Age category**				

(15–16)	Ref	–	–	–

Age groups (17–19)	.12	0.81	–.84	1.07

**Viral load**				

Suppression	Ref			

Suppressed	–1.03	0.034	–1.98	–.77


## Discussion

The study explored service use, frequency, satisfaction, risk and resilience among ALHIV aged 15–19 attending an ART clinic and TCC. ALHIV used formal and alternative services, as available to them, to do well despite adversity. Health workers and PMKs – adults who knew a lot about ALHIV lives — supplemented the data on service use experiences and offered further perspectives. All indicated that ALHIV used three or more services, including the ART clinic, teen-club clinic, community-based teen-clubs, YFHS and schools. PMKs, including biological parents and other caregivers, mentioned traditional and spiritual support to enhance ALHIV wellbeing. Using health workers and PMKs to negotiate resources supported the notion that both relational and institutional ecologies are key in sustaining ALHIV wellbeing. Multiple perspectives are crucial to informing integrated service provision and plans.

ALHIV frequently used institutional support, both ART clinics and TCC. Consistent with other research on the potential benefits of adolescent-centred approaches [64,65], participants (369/406, 91%) were especially satisfied with the TCC and its comprehensive programmes and accommodation of health workers. Service use frequency had statistically significant direct effects on service user satisfaction. Our qualitative data analysis extended our understanding of this, indicating that satisfactory service experience with TCC was integral to ALHIV wellbeing. This, in turn, ensured better clinical results (i.e. reduced viral load) and enhanced support from health workers. Previous studies on how health workers facilitate resilience have been disappointing [39,65]. In our study, health workers in teen-club settings played a double role as a provider and mentor and were able to form positive and caring relationships with ALHIV. The shift away from a paternalistic focus on health worker relationships with ALHIV is critical to developing autonomy, informed choices and decision making among ALHIV [66]. In a positive clinic environment, with comprehensive and ‘bundled programmes’, ALHIV felt a sense of belonging and felt in control of decision-making, contributing to resilience.

Prioritising the ART clinic and TCC among ALHIV is not unexpected given their health needs and the DSD policy drives to provide patient-centred care [66,67]. In our study context, health workers provided extensive psychosocial support to ensure ALHIV contributed to how services were delivered, that they understood them, and experienced a sense of belonging and satisfaction over time. Previous studies in Malawi showed that TCC attendance had the potential to reduce attrition and improve viral load suppression, hence, the salience of health interventions and YFHS among key populations [37,68]. Our study indicates that ALHIV with suppressed viral load had a lower score of service use as they were satisfied with the TCC. ALHIV appreciated the respect accorded to them in the clinic and role-play activities and the support and interactions which strengthened relationship building. These factors appeared to support not only access and adherence to treatment but encouraged self-care, effective communication and participation [69,70].

Sociodemographic variables and, service use, were statistically significant with respect to resilience in our study. Previous studies show that older youths, young women, and adolescents, generally report higher levels of service use and moderate resilience scores [8,71] compared with younger adolescents and males. In our study, adolescents aged 17–19 and most ALHIV sought services elsewhere and recorded moderate resilience scores. The mean resilience score for ALHIV in Malawi at 118.2 is consistent with other key populations [72]. In addition, young men and all out-of-school youth reported greater service utilisation, suggesting their capacity for positive adaptation regardless of life circumstances. This is consistent with a report on YFHS in Malawi, which observed that older and male adolescents strived for autonomy and greater independence, creating space for better decision making for better service use [65].

The unique aspects of our study focused on promotive factors, direct, indirect and total effects, and their significance on resilience. The interaction between service use frequency, satisfaction, peer risks, internal and external community risks are key in influencing resilience. However, we view resilience not as a final accomplished outcome, but as a dynamic and complicated process. In the current study, individual dimensions were connected with social and contextual influences as part of the multiple variables previously found to be associated with resilience [73,74]. The interaction of resources within and externally, and continued engagement with services, enhance resilience for ALHIV. These findings inform our understanding of the interaction of complex factors to ensure resilience in different situations and over time [7,75].

While increased service use does not necessarily mean better health or social outcomes, female ALHIV used limited services [50,65], reinforcing the understanding that service availability does not translate into utilisation [71,76]. The comprehensiveness of activities and improved quality of services enhanced service use satisfaction [69]. SEM and regression analysis demonstrated positive and direct effects between risks, service usage, frequency, satisfaction and resilience, but there was no association between resilience and viral load suppression. Given the increased vulnerability of ALHIV, particularly females, future studies are needed to examine resilience-promoting spaces and places within adolescent-centred TCC models to enable equitable access, provision to services and supports.

## Lessons learned

A few policy and practice lessons can be learned from this study, particularly after triangulating, summarising and interpreting multiple perspectives, feedback sessions and experiences involving adolescents, health care worker and caregivers. At the policy level, the teen-club clinic model facilitates differentiated care in relation to gender, age and clinical outcomes. The model paves the way for active interactions and equal partnerships between ALHIV, health workers and parents/caregivers. This adolescent-centred approach not only offers autonomy and allows ALHIV to voice their needs but ensures the one-stop-shop caters for the provision of services by specialized health workers in different rooms, facilitating internal and external support.

In practice, ALHIV service use and care pathways are complex and require teamwork and integration of various supports and relationships across care settings. ALHIV engaging in informed and shared decision-making for HIV care and other social and health matters are seen as ‘agentic beings’ thriving for their wellbeing. As part of optimizing access and service use, tailor-made HIV programmes are needed to meet multiple ALHIV needs, including counselling, psychosocial services, and transport and education allowances. The implications of a more integrated provision of support for ALHIV is likely to encourage a bi-directional referral between services and improved monitoring and evaluation at facility levels. This collaborative effort may improve health outcomes, empower adolescent-health care worker relationships and management of support services. Overall, a well-coordinated and integrated service delivery strengthens adolescents centered care experiences and broadens their intersectoral support and options for social and health services.

## Limitations

Our study was cross-sectional, within a single facility in Malawi, which makes it difficult to determine the causal processes and external validity. We depended on ALHIV self-reports on service use histories and support in the past year; their experiences of and access to formal and alternatives services may be unreliable because of recall bias [51]. Working with ALHIV and attending UFC ART clinic and TCC may also have caused selection bias. The low turnout by parents and caregivers as PMKs made it difficult to link their data with ALHIV. Lack of completion on entries in the registers and EMR database also provided challenges in defining relationships, viral load, clinic registration, and attendance sheet outcomes. However, multiple sources of data and perspectives using qualitative and quantitative methods provided complementary information and allowed a deeper understanding of service use and resilience-related outcomes.

## Conclusion

A mixture of health-related services (ART, teen-club clinic, YFHS), school and community-based teen-clubs form an integral part of ALHIV formal service provision; satisfaction with these services is associated with resilience. In addition to formal service use, most PMKs regarded traditional and spiritual care as alternative supports to ensure a holistic provision of care for ALHIV. There were significant associations between sociodemographic variables and resilience, and positive and indirect effects on the same, supporting the idea of services nurturing ALHIV wellbeing. The adolescent-centred approach made it possible to promote service use which helped ALHIV beyond the clinical care.

Resilience is a complex construct. Its supportive value depends on multiple support systems and processes that include individual autonomy and independence, typical and atypical relational maintenance, and institutional and policy support. The qualitative findings strengthened our understanding of the relationship between service use satisfaction, including enhancing access to care, participation, and empowering ALHIV and caregivers within the TCC. The multiple roles of health workers in partnership with parents/caregivers encouraged adherence to medication, provided psychosocial support and monitored viral load, all of which fostered resilience and reduced adversity. The TCC setting offers comprehensive activities and ultimately promotes resilience and wellbeing among ALHIV. Thus, TCC is an example of a core package of interventions that combine evidence-based approaches that go beyond the health sector.
